# Effects of dietary mulberry leaves on growth, production performance, gut microbiota, and immunological parameters in poultry and livestock: a systematic review and meta-analysis

**DOI:** 10.5713/ab.23.0449

**Published:** 2024-02-28

**Authors:** Bing Geng, Jinbo Gao, Hongbing Cheng, Guang Guo, Zhaohong Wang

**Affiliations:** 1 Shandong Institute of Sericulture, Yantai 264001, China; 2 Yantai Key Laboratory, Yantai, 264001, China; 3 Shandong Engineering Technology Research Center, Yantai, 264001, China

**Keywords:** Growth, Gut Microbiota, Meta-analysis, Mulberry Leaves, Poultry and Livestock, Production Performance

## Abstract

**Objective:**

This study aimed to assess the effects of dietary mulberry leaves on the growth, production performance, gut microbiota, and immunological parameters of poultry and livestock.

**Methods:**

The PubMed, Embase, and Scopus databases were systematically analyzed to identify pertinent studies up to December 2022. The effects of mulberry leaf diet was assessed using the weighted mean difference, and the 95% confidence interval was calculated using a random-effects model.

**Results:**

In total, 18 studies that sampled 2,335 poultry and livestock were selected for analysis. Mulberry leaves improved the average daily gain and reduced the feed/meat ratio in finishing pigs, and the average daily gain and average daily feed intake in chicken. In production performance, mulberry leaves lowered the half carcass weight, slaughter rate, and loin eye area in pigs, and the slaughter rate in chickens. Regarding meat quality in pigs, mulberry leaves reduced the cooked meat percentage, shear force, crude protein, and crude ash, and increased the 24 h pH and water content. In chickens, it increased the drip loss, shear force, 45 min and 24 h pH, crude protein, and crude ash. Mulberry leaves also affect the abundances of gut microbiota, including *Bacteroides*, *Prevotella*, *Megamonas*, *Escherichia-Shigella*, *Butyricicoccus*, unclassified *Ruminococcaceae*, *Bifidobacterium*, *Lactobacillus*, and *Escherichia coli* in poultry and livestock. Mulberry leaves at different doses were associated with changes in antioxidant capacity in chickens, and immune organ indexes in pigs. With respect to egg quality, mulberry leaves at different doses improved the shell strength, yolk color, eggshell thickness, and eggshell weight. However, moderate doses diminished the egg yolk ratio and the egg yolk moisture content.

**Conclusion:**

In general, dietary mulberry leaves improved the growth, production performance, and immunological parameters in poultry and livestock, although the effects varied at different doses.

## INTRODUCTION

The increasing demand for animal products necessitates the acquisition of economically viable livestock feed alternatives. Thus, it is imperative to identify cost-effective alternative feeds to facilitate the development of animal husbandry. Moreover, the shortage of animal feed has resulted in the escalation of their market prices. Several studies have already demonstrated the potential of utilizing unconventional feed to replace cereal-based feed, while ensuring minimal impact on animal production performance [1,2]. Moreover, unconventional feed can have additional benefits such as improving the gut microbiota and other immunological parameters. Therefore, it is particularly important to find inexpensive yet beneficial feed alternatives that can replace conventional animal feed [3].

The mulberry plant (*Morus alba*) is a fast-growing shrub, and its leaves are utilized as the primary food source for silkworms [4]. In China, the cultivation of mulberry is estimated to cover 700,000 ha, yielding nearly 18,200,000 tons [5]. For herbivores, mulberry leaves are highly palatable and rich in digestible macronutrients, with a high protein content and essential amino acid profile [6]. Moreover, the protein content of mulberry leaves is higher than those in traditional forages, making them a potential high-quality protein source for livestock [7]. Fresh mulberry leaves can be directly incorporated into the diet of animals, whereas ensiled mulberry leaves can be fed to bovine animals, with no adverse effects to their performance and carcass quality [8,9]. In addition, mulberry leaves are an excellent protein-rich forage for both monogastric and ruminant animals owing to the leaf’s unique nutrient profile, digestibility, and palatability [10]. Moreover, the active components of mulberry leaves can also help regulate the antioxidant capacity of laying hens and the antioxidative profile and lipid metabolism of pigs [11,12].

Several studies have investigated the effects of a mulberry leaf diet on various poultry and livestock, however, their results have been inconsistent [11,13–29]. Moreover, no systematic review has yet summarized the effects of mulberry leaves on the growth, production performance, gut microbiota, and immunological parameters of poultry and livestock. Therefore, this study conducted a systematic review and meta-analysis to provide both the qualitative and the quantitative results regarding the effects of a mulberry leaf diet on poultry and livestock production.

## MATERIALS AND METHODS

### Data sources, search strategy, and selection criteria

The Preferred Reporting Items for Systematic Reviews and Meta-Analyses checklist was used to guide this systematic review and meta-analysis [30]. Studies that reported the effects of mulberry leaves on poultry and livestock were eligible for our study, irrespective of the publication language and status. We systematically searched the PubMed, Embase, and Scopus databases up until December 2022 using the following terms: ‘mulberry leaf,’ ‘mulberry leaves,’ ‘Morus indica L,’ ‘1-deoxynojirimycin,’ and ‘1-DNJ’. We also reviewed the reference lists of relevant articles and reviews to identify any potentially eligible studies.

The literature search and study selection were independently performed by two reviewers, and any disagreements between the reviewers were resolved by mutual discussion until a consensus was reached. Studies were eligible if they met the following criteria: i) animals: poultry and livestock; ii) intervention: mulberry leaves of various types and dosing; iii) control: basal diet; iv) outcome: growth performance, production performance, meat quality, gut microbiota, serum antioxidant capacity, immunological parameters, and egg quality; and v) study design: intervention study. The exclusion criteria included: i) studies that did not utilize mulberry leaves; ii) other interfering factors or the use of multi-component interventions; and iii) studies that did not report data regarding growth, production performance, gut microbiota, and immunological parameters.

### Data collection and quality assessment

The data collection was also independently performed by two reviewers. The abstract information included the first author’s surname, publication year, region, sample size, age, type of animal, intervention, and control, and investigated outcomes. Study quality was also assessed using the Jadad scale [31] based on randomization, blinding, allocation concealment, withdrawals and dropouts, and intention-to-treat analysis to assess the methodological quality of the eligible studies. Studies with a score of 4 or 5 were considered as high quality. Inconsistent results between the reviewers for data collection and quality assessment were resolved by a third reviewer who referred to the full text of the article.

### Statistical analyses

All investigated outcomes were assigned as continuous variables, and weighted mean differences (WMD), and the 95% confidence intervals (CIs) were calculated before data pooling. Subsequently, the pooled effect estimates were calculated using a random-effects model that considered the underlying variation across the included studies [32,33]. Heterogeneity among the included studies was assessed using *I*^2^ and Q statistics, and significant heterogeneity was defined as *I*^2^> 50.0% or p<0.10 [34,35]. The p-value for the pooled results was 2-sided, with the inspection level set at 0.05. Statistical analyses were performed using STATA version 14.0 (Stata Corporation, College Station, TX, USA).

## RESULTS

### Literature search

**Figure 1:**
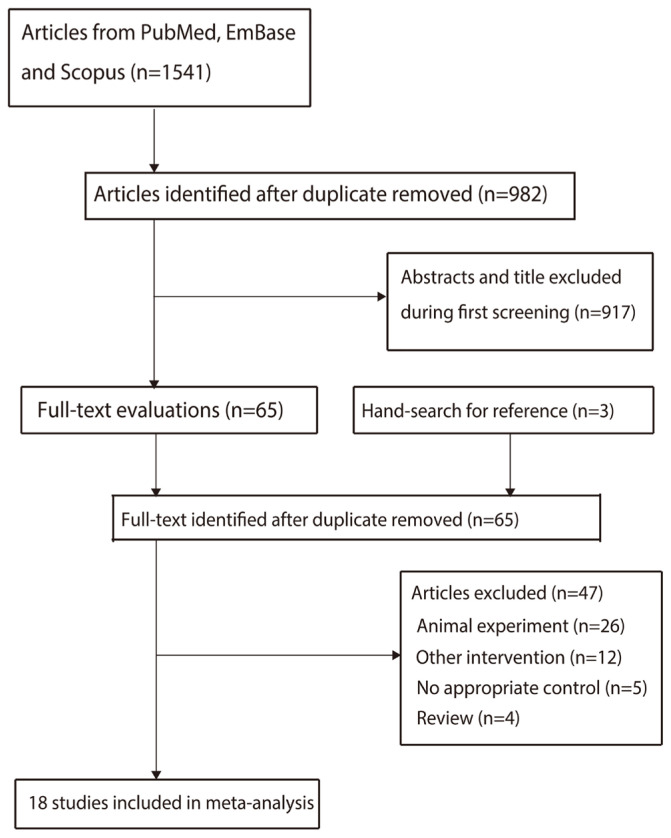
The details of literature search and study selection process.

The initial electronic search yielded 1,541 articles, which were filtered to 982 articles after removing the duplicates. A further 917 articles were excluded since they reported irrelevant topics. The remaining 65 studies were then retrieved for full-text evaluation, with 47 studies being further excluded as they included animal experiments (n = 26), other interventions (n = 12), no appropriate control (n = 5), and reviews (n = 4). Additionally, no new eligible studies were identified by reviewing the reference lists of relevant articles. Finally, a total of 18 studies were included in the meta-analysis [11,13–29] (Figure 1).

### Study characteristics

The baseline characteristics of the studies that met the final eligibility criteria are summarized in Table 1. A total of 2,335 poultry and livestock samples were included in these studies, with the sample sizes ranging from 18 to 360. Of these studies, 17 were performed in China, while one was conducted in Thailand. Six studies investigated the effects of mulberry leaves on chickens, seven on pigs, and the remaining five on other poultry and livestock. All studies mentioned randomization, withdrawals, and dropouts, and the details of the methodological quality of the selected studies are shown in Table 2.

### Growth performance

A summary of the effects of mulberry leaves on growth performance is shown in Table 3. A low dose of mulberry leaves was associated with an elevated average daily gain (WMD, 5.30; 95%CI, 4.79 to 5.81; p<0.001), and an average daily feed intake (WMD, 3.60; 95%CI, 2.60 to 4.60; p<0.001). Meanwhile, a moderate dose of mulberry leaves significantly increased average daily gain (WMD, 92.00; 95%CI, 61.68 to 122.32; p<0.001) and reduced feed/meat ratio (WMD, −0.20; 95%CI, −0.34 to −0.06; p = 0.006) in pigs. In addition, a moderate dose positively influenced the average daily gain in both pigs (WMD, 2.80; 95%CI, 2.27 to 3.33; p<0.001) and chickens (WMD, 5.20; 95%CI, 3.89 to 6.51; p<0.001). Furthermore, a high dose of mulberry leaves in chickens was associated with an increase in the average daily gain (WMD, 2.00; 95%CI, 1.47 to 2.53; p<0.001) and the average daily feed intake (WMD, 2.00; 95%CI, 0.91 to 3.09; p<0.001).

### Production performance

A summary of the effects of mulberry leaves on production performance is shown in Table 4. First, a low dose of mulberry leaves was associated with low slaughter rates in pigs (WMD, −1.19; 95%CI, −1.48 to −0.90; p<0.001) and chickens (WMD, −1.50; 95%CI: −1.68 to −1.32; p<0.001). Second, a moderate dose of mulberry leaves significantly reduced slaughter rates (WMD, −0.71; 95%CI, −1.24 to −0.18; p = 0.009) and increased the loin eye area (WMD, 5.00; 95%CI, 0.87 to 9.13; p = 0.018) in pigs. In chickens, a moderate dose of mulberry leaves was associated with low slaughter rates (WMD, −1.10; 95%CI, −1.31 to −0.89; p<0.001). Finally, a high dose of mulberry leaves was associated with low half carcass weight (WMD, −2.72; 95%CI, −3.57 to −1.86; p<0.001) and slaughter rates (WMD, −2.74; 95%CI, −4.62 to −0.86; p = 0.004) for pigs. In chickens, a high dose of mulberry leaves reduced the slaughter rates (WMD, −1.10; 95%CI, −1.29 to −0.91; p<0.001).

### Meat quality

A summary of the effects of mulberry leaf extract on meat quality is shown in Table 5. First, a low dose of mulberry leaves was associated with a low cooked meat percentage (WMD, −3.56; 95%CI, −5.47 to −1.65; p<0.001), and crude ash (WMD, −0.28; 95%CI, −0.49 to−0.07; p = 0.008) in pigs. In chickens, a low dose of mulberry leaves affected the drip loss (WMD, −0.92; 95%CI: −1.05 to −0.79; p<0.001), shear force (WMD, 0.09; 95%CI, 0.03 to 0.15; p = 0.002), 45 min pH (WMD, 0.26; 95%CI, 0.24 to 0.28; p<0.001), 24 h pH (WMD, −0.05; 95%CI, −0.08 to −0.02; p = 0.002), crude protein (WMD, 7.10; 95%CI, 6.53 to 7.67; p<0.001), and crude ash (WMD, 1.90; 95%CI, 0.50 to 3.30; p = 0.008). Second, a moderate dose of mulberry leaves significantly reduced cooked meat percentage (WMD, −2.37; 95%CI, −4.49 to −0.25; p = 0.029), shear force (WMD, −23.02; 95%CI, −41.71 to −4.33; p = 0.016), and crude ash (WMD, −0.58; 95%CI, −1.02 to −0.14; p = 0.009) and alternatively increased the 24 h pH (WMD, 0.24; 95%CI, 0.10 to 0.38; p = 0.001) and water content (WMD, 0.61; 95%CI, 0.18 to 1.04; p = 0.006) in pigs. In chickens, a moderate dose of mulberry leaves affected the drip loss (WMD, −0.65; 95%CI, −0.80 to −0.50; p<0.001), shear force (WMD, −0.11; 95%CI, −0.16 to −0.06; p<0.001), 45 min pH (WMD, 0.23; 95%CI, 0.20 to 0.26; p<0.001), 24 h pH (WMD, 0.09; 95%CI, 0.06 to 0.12; p<0.001), and crude protein (WMD, 4.50; 95%CI, 3.99 to 5.01; p<0.001). Finally, a high dose of mulberry leaves was associated with lower shear force (WMD, −5.26; 95%CI, −6.68 to −3.83; p<0.001), crude protein (WMD, −0.63; 95%CI, −1.14 to −0.12; p = 0.016), or crude ash (WMD, −1.01; 95%CI, −1.56 to −0.46; p<0.001), and elevated 24 h pH (WMD, 0.16; 95%CI, 0.05 to 0.28; p = 0.005) and water content (WMD, 0.90; 95%CI, 0.25 to 1.55; p = 0.007) in pigs. In chickens, a high dose of mulberry leaves affected the drip loss (WMD, −0.87; 95%CI, −1.04 to −0.70; p<0.004), 45 min pH (WMD, 0.24; 95%CI, 0.22 to 0.26; p<0.001), crude protein (WMD, 5.10; 95%CI, 4.57 to 5.63; p<0.001), and crude ash (WMD, 3.60; 95%CI, 2.46 to 4.74; p<0.001).

### Gut microbiota

The results of the gut microbiota analysis were qualitatively summarized. Chen et al [13] previously found that mulberry leaves enhanced the abundance of *Bacteroides*, *Prevotella*, and *Megamonas* in chicken guts. Additionally in geese, Hou et al [18] found that 1-deoxynojirimycin from mulberry leaves was associated with an increased abundance of *Bacteroides*, *Escherichia-Shigella*, and *Butyricicoccus*, whereas the abundance of unclassified *Ruminococcaceae* was reduced. Furthermore, Song et al [29] indicated that the use of mulberry leaf extract was associated with an elevated number of beneficial bacteria, such as *Bifidobacterium* and *Lactobacillus*, whereas the presence of the potentially pathogenic bacterium *Escherichia coli* was reduced in the colon on piglets.

### Serum antioxidant capacity and immune organ indexes

A summary of the effects of mulberry leaves on serum antioxidant capacity and immune organ indices in pigs is shown in Table 6. We noted that low (WMD, 0.82; 95%CI, 0.40 to 1.24; p<0.001), moderate (WMD, 1.62; 95%CI, 1.23 to 2.01; p<0.001), and high (WMD, 1.85; 95%CI, 1.39 to 2.31; p<0.001) doses of mulberry leaves were associated with elevated catalase activity. Additionally, low (WMD, 0.14; 95%CI, 0.00 to 0.28; p = 0.043), moderate (WMD, 0.48; 95%CI, 0.35 to 0.61; p<0.001), and high (WMD, 0.21; 95%CI, 0.07 to 0.35; p = 0.003) doses of mulberry leaves significantly increased total antioxidant capacity. Low (WMD, 3.91; 95%CI, 0.67 to 7.15; p = 0.018), moderate (WMD, 9.68; 95%CI, 7.01 to 12.35; p<0.001), and high (WMD, 14.22; 95%CI, 11.03 to 17.41; p<0.001) doses of mulberry leaves were also associated with an elevated superoxide dismutase. Furthermore, low (WMD, 7.38; 95%CI, 3.94 to 10.82; p<0.001), moderate (WMD, 17.89; 95%CI, 14.07 to 21.71; p<0.001), and high (WMD, 21.89; 95%CI, 18.24 to 25.54; p<0.001) doses of mulberry leaves significantly increased glutathione peroxidase. However, low (WMD, −0.72; 95%CI, −1.19 to −0.25; p = 0.003), moderate (WMD, −1.45; 95%CI, −1.92 to −0.98; p<0.001), and high (WMD, −1.71; 95%CI, −2.29 to −1.13; p<0.001) doses of mulberry leaves all significantly reduced malondialdehyde. In addition, we noted that low (WMD, 0.35; 95%CI, 0.05 to 0.65; p = 0.024) and high (WMD, 0.56; 95%CI, 0.15 to 0.97; p = 0.008) doses of mulberry leaves were associated with an increased thymus index. Moreover, low (WMD, 0.21; 95%CI, 0.06 to 0.36; p = 0.005), and high (WMD, 0.21; 95%CI, 0.07 to 0.35; p = 0.004) doses of mulberry leaves had no significant effects on the spleen index. A high dose of mulberry leaves was also associated with lower immunoglobulin (Ig) M (WMD, −0.09; 95%CI, −0.15 to −0.03; p = 0.003). Whereas low doses of mulberry leaves were associated with an increased IgG (WMD, 1.01; 95%CI, 0.47 to 1.55; p<0.001). Lastly, both low and high doses of mulberry leaves were correlated with elevated levels of interleukin (IL)-1β, IL-2, IL-6, IL-8, and interferon (INF)-γ.

### Egg quality

A summary of the effects of mulberry leaf extract on egg quality in chickens is shown in Table 7. Low (WMD, 2.91; 95%CI, 1.31 to 4.51; p<0.001), moderate (WMD, 3.52; 95%CI, 1.85 to 5.19; p<0.001), and high (WMD, 4.85; 95%CI, 3.24 to 6.46; p<0.001) doses of mulberry leaves significantly increased eggshell strength. Low (WMD, 0.61; 95%CI, 0.32 to 0.90; p<0.001), moderate (WMD, 0.77; 95%CI, 0.47 to 1.07; p<0.001), and high (WMD, 0.94; 95%CI, 0.71 to 1.17; p< 0.001) doses of mulberry leaves were associated with elevated yolk color. In addition, low (WMD, 0.02; 95%CI, 0.01 to 0.03; p<0.001) and moderate (WMD, 0.02; 95%CI, 0.01 to 0.03; p<0.001) doses of mulberry leaves significantly increased eggshell thickness. Notably, moderate doses of mulberry leaves were associated with increased eggshell weight (WMD, 0.21; 95%CI, 0.02 to 0.40; p = 0.032). However, low (WMD, −1.34; 95%CI, −1.65 to −1.03; p<0.001), moderate (WMD, −0.69; 95%CI, −1.01 to −0.37; p<0.001), and high (WMD, −0.52; 95%CI, −0.81 to −0.23; p<0.001) doses of mulberry leaves were all also associated with a reduced egg yolk ratio. Lastly, moderate (WMD, −1.82; 95%CI, −3.43 to −0.21; p = 0.026) and high (WMD, −4.43; 95%CI, −6.71 to −2.15; p< 0.001) doses of mulberry leaves significantly reduced the egg yolk moisture content.

## DISCUSSION

Although mulberry leaves are widely used in poultry and livestock, their effects on growth, production performance, gut microbiota, and immunological parameters remain unclear. This systematic review and meta-analysis investigated the effects of mulberry leaf extract on poultry and livestock. A total of 2,335 poultry and livestock samples from 18 studies with a wide range of interventions were utilized in this review. Regarding growth performance, we noted that a moderate dose of mulberry leaves increased the average daily gain while reducing the feed/meat ratio in pigs, whereas mulberry leaves significantly increased the average daily gain and average daily feed intake in chickens. We noted that mulberry leaves could affect half the carcass weight, slaughter rate, and loin eye area in pigs and reduced the slaughter rate in chickens. Regarding meat quality, we additionally noted that cooked meat percentage, shear force, 24 h pH, crude protein, water, and crude ash in pigs could be all affected by mulberry leaves, whereas mulberry leaves could also impact drip loss, shear force, 45 min pH, 24 h pH, crude protein, and crude ash in chickens. Regarding serum antioxidant capacity and immune organ indices, we noted that the thymus index, spleen index, IgM, and IgG were all affected by mulberry leaves in pigs. Whereas, the serum antioxidant capacity of catalase activity, total antioxidant capacity, superoxide dismutase, and glutathione peroxidase were all elevated in chicken receiving mulberry leaves, except for malondialdehyde, which reduced after receiving mulberry leaves. The inflammatory indexes of IL-1β, IL-2, IL-6, IL-8, and INF-γ in pigs were all significantly increased after using mulberry leaves. Mulberry leaves also affected eggshell strength, yolk color, eggshell thickness, eggshell weight, egg yolk ratio, and egg yolk moisture content.

Our study ultimately found that the use of mulberry leaves increased the average daily gain and feed/meat ratio in pigs, and the average daily gain and average daily feed intake of chickens. Mulberry leaves have been reported to improve muscle formation and development in pigs by modulating the expression of various genes, including *ACOT4*, *ECHS1*, *HACD1*, *NPR1*, *ADCY2*, *MGLL*, and *IRS1*, which could affect fatty acid metabolism. Muscle formation and development could be affected by *TNNC1*, *TNNT1*, and *MYL3* [14]. Moreover, growth performance could be improved by nutrient digestion and absorption, while the use of mulberry leaves could improve the digestibility of dry matter [36]. In addition, we noted that mulberry leaves could reduce half-carcass weight, slaughter rate, and loin eye area in pigs, while the use of mulberry leaves in chickens could reduce the slaughter rate. Several reasons could explain these results: i) mulberry leaves contained less crude fiber and neutral detergent fiber; thus, the nutrients were easier to digest and absorb, resulting in superior carcass traits [20]; and ii) the slaughter rate in chickens was reduced after mulberry leaves were used, which was significantly related to growth performance.

Collectively, the results highlight that mulberry leaves could affect the cooked meat percentage, shear force, 24 h pH, crude protein, water, and crude ash in pigs. Moreover, the use of mulberry leaf was significantly associated with drip loss, shear force, 45 min pH, 24 h pH, crude protein, and crude ash in chickens. This can be attributed to the heightened activity of intestinal amylase resulting from the use of mulberry leaves. As the activity of intestinal digestive enzymes can improve after using mulberry leaves, it can subsequently improve the growth, nutrient digestion, or absorption in animals [37].

We noted that the inflammatory indices, thymus index, spleen index, IgM, and IgG in pigs could all be affected by mulberry leaves. Mulberry leaves have been reported to improve the digestion and absorption of nutrients, enhance immunity and disease resistance, and inhibit metabolic processes, thus increasing the growth performance of animals [38,39]. Furthermore, mulberry leaves presented immunomodulating activity, which could affect varies kind cells and macrophage-dependent immune system responses [40]. In addition, Mullberry leaves have also been observed to alleviate inflammation via interactions among insulin signaling pathway and TNF-α [41]. In chickens, the eggshell strength, yolk color, eggshell thickness, eggshell weight, egg yolk ratio, and egg yolk moisture content were all affected by mulberry leaves. The improved eggshell strength can be attributed to mulberry leaf flavonoids that increase the antioxidant capacity of the uterine shell gland and calcium deposition [27]. Similarly, the dark yolk color, which is significantly related to better egg quality, could be attributed to the carotenoid content of mulberry leaves [42].

However, this study noted several limitations. First, the basal diet differed across the included studies, which may have affected the observed effects of mulberry leaves. Second, the analysis included various poultry and livestock, with the characteristics of the animals differing, which could have influenced the effects of mulberry leaves. Third, the final dataset included a relatively small number of studies, which could affect the reliability of the pooled conclusion. Lastly, the analysis was based on published articles with the inherent limitations of inevitable publication bias and restricted detailed analysis.

Notably, this study is the first to summarize the effects of mulberry leaves on the growth, production performance, gut microbiota, and immunological parameters of poultry and livestock. In pigs, mulberry leaves affect positively influenced parameters like average daily gain, feed/meat ratio, half-carcass weight, and crude protein, water, and crude ash levels, while lowering the carcass weight and slaughter rates. They also enhance certain aspects of meat quality, such as loin eye area and cooked meat percentage. However, mulberry leaves were also shown to alter the shear force, pH levels, thymus index, spleen index, and immunoglobulins (IgM and IgG). Conversely, in chicken, the use of mulberry leaves positively affected the average daily gain, egg quality attributes like eggshell strength, yolk color, eggshell thickness, and eggshell weight, while also affecting variables such as feed intake, slaughter rate, drip loss, and pH levels. However, mulberry leaves also reduced the egg yolk ratio and moisture content. Nevertheless, the meta-analysis revealed mulberry leaves as potential alternative feed, with both advantages and considerations, for poultry and livestock management.

## Figures and Tables

**Table 1:** The baseline characteristics of included studies and involved poultries and livestock

Study	Region	Sample size	Age	Animal species	Intervention
Lin 2017 [11]	China	96	22 wk	Laying hens [HENDRIX]	Basal diet; 0.5% MLP plus basal diet; 1% MLP plus basal diet; and 2% MLP plus basal diet
Chen 2019 [13]	China	100	20 d	Chickens	Basal diet plus 4% MLP; basal diet
Chen 2019 [14]	China	56	35 d	Landrace-Yorkshire hybrid pigs	Basal diet; 3% MLP plus basal diet; 6% MLP plus basal diet; and 9% MLP plus basal diet
Zhao 2019 [15]	China	120	28 d	Duroc-Landrace-Yorkshire crossbred weanling pigs	Basal diet; 0.6 g/kg MLP plus basal diet; 1.2 g/kg MLP plus basal diet
Liu 2019 [16]	China	180	NA	Finishing barrows [Xiangcun Black pigs]	Basal diet; 3% MLP plus basal diet; 6% MLP plus basal diet; 9% MLP plus basal diet, and 12% MLP plus basal diet
Li 2020 [17]	China	40	90 d	Multiparous Murrah buffaloes	Basal diet; 15 g/d MLF plus basal diet; 30 g/d MLF plus basal diet; and 45 g/d MLP plus basal diet
Hou 2020 [18]	China	128	1 2 d	Wanxi white geese	Basal diet; 0.05 mg/g DNJ plus basal diet; 0.1 mg/g DNJ plus basal diet; and 0.15 mg/g DNJ plus basal diet
Sun 2020 [19]	China	32	4.0 mon	Small-tailed Han sheep	Basal diet; 8% MLP plus basal diet; 24% MLP plus basal diet; and 32% MLP plus basal diet
Chen 2021 [20]	China	117	NA	Duroc-Landrace-Yorkshire crossbred weanling pigs	Basal diet; and 4% MLP plus basal diet
Ding 2021 [21]	China	360	1.0 d	Chicken broilers	Basal diet; 3% MLP plus basal diet; 6% MLP plus basal diet; and 9% MLP plus basal diet
So-In 2022 [22]	Thailand	276	3.0 wk	Chicken broilers	Basal diet; 10% MLP plus basal diet
Zhang 2022 [23]	China	288	38 wk	Laying hens [Lohmann Silber]	Basal diet; 0.4% MLE plus basal diet; 0.8% MLE plus basal diet; and 1.2% MLE plus basal diet
Wang 2022 [24]	China	18	6.0 mon	Tibetan pigs	Basal diet; and 8% MLP plus basal diet
Ma 2022 [25]	China	120	NA	Landrace-Yorkshire hybrid pigs	Basal diet; 6% MLP plus basal diet; 9% MLP plus basal diet; and 12% MLP plus basal diet
Long 2022 [26]	China	44	5.0–6.0 mon	Guizhou crossbred black goats	Basal diet; 40% MLP plus basal diet
Huang 2022 [27]	China	270	60.0 wk	Qiling breeder hens	Basal diet; 30 mg/kg MLF plus basal diet; 60 mg/kg MLF plus basal diet
Yang 2022 [28]	China	50	NA	Xiangcun Black sows	Basal diet; 100 g MLP plus basal diet; 200 g MLP plus basal diet; 300 g MLP plus basal diet; and 400 g MLP plus basal diet
Song 2022 [29]	China	40	28.0 d	Duroc-Landrace-Yorkshire crossbred weanling piglets	Basal diet; and 0.1% MLE plus basal diet

MLP, mulberry leaf powders; DNJ, deoxynojirimycin; MLF, mulberry leaf flavonoids; MLE, mulberry leaf extract; NA, not available.

**Table 2:** The details of methodological quality of included studies

Study	Randomization	Blinding	Allocation concealment	Withdrawals and dropouts	Intention-to-treat analysis	Overall
Lin 2017 [11]	1	1	0	1	0	3
Chen 2019 [13]	1	1	0	1	1	4
Chen 2019 [14]	1	0	0	1	1	3
Zhao 2019 [15]	1	0	0	1	1	3
Liu 2019 [16]	1	1	0	1	1	4
Li 2020 [17]	1	1	0	1	1	4
Hou 2020 [18]	1	1	0	1	1	4
Sun 2020 [19]	1	1	0	1	1	4
Chen 2021 [20]	1	1	0	1	1	4
Ding 2021 [21]	1	1	0	1	0	3
So-In 2022 [22]	1	1	0	1	0	3
Zhang 2022 [23]	1	0	0	1	0	2
Wang 2022 [24]	1	0	0	1	1	3
Ma 2022 [25]	1	0	0	1	1	3
Long 2022 [26]	1	1	0	1	0	3
Huang 2022 [27]	1	0	0	1	0	2
Yang 2022 [28]	1	1	0	1	1	4
Song 2022 [29]	1	0	0	1	1	3

**Table 3:** The summary results of mulberry leaf powders on growth performance

Outcome	Animal species	Intervention	No of studies	WMD and 95%CI	p-value	*I* ^2^	Q statistic
Average daily gain	Pigs	Low	3	14.44 (−21.67 to 50.55)	0.433	82.8	0.001
		Moderate	1	92.00 (61.68 to 122.32)	<0.001	-	-
		High	3	11.64 (−40.04 to 63.33)	0.659	96.7	<0.001
	Chicken	Low	1	5.30 (4.79 to 5.81)	<0.001	-	-
		Moderate	1	2.80 (2.27 to 3.33)	<0.001	-	-
		High	1	2.00 (1.47 to 2.53)	<0.001	-	-
Average daily feed intake	Pigs	Low	3	0.02 (−0.10 to 0.13)	0.755	90.0	<0.001
		Moderate	1	0.08 (−0.17 to 0.33)	0.523	-	-
		High	3	−0.02 (−0.06 to 0.01)	0.198	65.2	0.057
	Chicken	Low	1	3.60 (2.60 to 4.60)	<0.001	-	-
		Moderate	1	5.20 (3.89 to 6.51)	<0.001	-	-
		High	1	2.00 (0.91 to 3.09)	<0.001	-	-
Feed:meat ratio	Pigs	Low	3	−0.10 (−0.21 to 0.01)	0.070	62.5	0.046
		Moderate	1	−0.20 (−0.34 to −0.06)	0.006	-	-
		High	3	0.14 (−0.15 to 0.43)	0.339	95.4	<0.001
	Chicken	Low	1	−0.20 (−0.83 to 0.43)	0.538	0.0	0.810

WMD, mean differences; CI, confidence interval.

**Table 4:** The summary results of mulberry leaf powders on production performance

Outcome	Animal species	Intervention	No of studies	WMD and 95%CI	p-value	*I* ^2^	Q statistic
Half carcass weight	Pigs	Low	2	1.31 (−6.04 to 8.67)	0.726	98.0	<0.001
		Moderate	1	−0.63 (−2.02 to 0.76)	0.373	-	-
		High	2	−2.72 (−3.57 to −1.86)	<0.001	0.0	0.599
Slaughter rate	Pigs	Low	1	−1.19 (−1.48 to −0.90)	<0.001	-	-
		Moderate	1	−0.71 (−1.24 to −0.18)	0.009	-	-
		High	1	−2.74 (−4.62 to −0.86)	0.004	-	-
	Chicken	Low	1	−1.50 (−1.68 to −1.32)	<0.001	-	-
		Moderate	1	−1.10 (−1.31 to −0.89)	<0.001	-	-
		High	1	−1.10 (−1.29 to −0.91)	<0.001	-	-
Average backfat thickness	Pigs	Low	2	−0.45 (−1.52 to 0.62)	0.411	0.0	0.843
		Moderate	1	−0.17 (−1.36 to 1.02)	0.780	-	-
		High	2	−2.24 (−6.51 to 2.03)	0.304	96.7	<0.001
Loin eye area	Pigs	Low	2	−0.73 (−6.11 to 4.65)	0.791	22.1	0.277
		Moderate	1	5.00 (0.87 to 9.13)	0.018	-	-
		High	1	−2.35 (−8.11 to 3.41)	0.424	-	-

WMD, mean differences; CI, confidence interval.

**Table 5:** The summary results of mulberry leaf powders on meat quality

Outcome	Animal species	Intervention	No of studies	WMD and 95%CI	p-value	*I* ^2^	Q statistic
Cooked meat percentage	Pigs	Low	1	−3.56 (−5.47 to −1.65)	<0.001	-	-
		Moderate	1	−2.37 (−4.49 to −0.25)	0.029	-	-
		High	1	−0.25 (−2.27 to 1.77)	0.808	-	-
Water loss rate	Pigs	Low	1	−0.03 (−1.34 to 1.28)	0.964	-	-
		Moderate	1	−0.80 (−1.75 to 0.15)	0.100	-	-
		High	1	−0.90 (−1.82 to 0.02)	0.055	-	-
Drip loss	Pigs	Low	2	−0.85 (−2.06 to 0.36)	0.167	78.7	0.009
		Moderate	1	0.24 (−3.07 to 3.55)	0.887	-	-
		High	1	−0.43 (−1.04 to 0.19)	0.173	0.0	0.950
	Chicken	Low	1	−0.92 (−1.05 to −0.79)	<0.001	-	-
		Moderate	1	−0.65 (−0.80 to −0.50)	<0.001	-	-
		High	1	−0.87 (−1.04 to −0.70)	<0.001	-	-
Shear force	Pigs	Low	2	−12.77 (−29.23 to 3.68)	0.128	0.0	0.899
		Moderate	1	−23.02 (−41.71 to −4.33)	0.016	-	-
		High	2	−5.26 (−6.68 to −3.83)	<0.001	0.0	0.323
	Chicken	Low	1	0.09 (0.03 to 0.15)	0.002	-	-
		Moderate	1	−0.11 (−0.16 to −0.06)	<0.001	-	-
		High	1	0.00 (−0.10 to 0.10)	1.000	-	-
Meat colour	Pigs	Low	1	0.57 (−0.24 to 1.38)	0.167	-	-
		Moderate	1	0.17 (−0.83 to 1.17)	0.740	-	-
		High	1	0.44 (−0.26 to 1.14)	0.216	-	-
PH (45 min)	Pigs	Low	2	0.03 (−0.07 to 0.14)	0.538	0.0	0.841
		Moderate	1	0.00 (−0.28 to 0.28)	1.000	-	-
		High	2	0.04 (−0.15 to 0.22)	0.688	43.5	0.183
	Chicken	Low	1	0.26 (0.24 to 0.28)	<0.001	-	-
		Moderate	1	0.23 (0.20 to 0.26)	<0.001	-	-
		High	1	0.24 (0.22 to 0.26)	<0.001	-	-
PH (24 hour)	Pigs	Low	2	0.06 (−0.19 to 0.31)	0.643	94.3	<0.001
		Moderate	1	0.24 (0.10 to 0.38)	0.001	-	-
		High	2	0.16 (0.05 to 0.28)	0.005	0.0	0.549
	Chicken	Low	1	−0.05 (−0.08 to −0.02)	0.002	-	-
		Moderate	1	0.09 (0.06 to 0.12)	<0.001	-	-
		High	1	−0.01 (−0.04 to 0.02)	0.565	-	-
Crude protein	Pigs	Low	2	0.41 (−1.12 to 1.94)	0.602	47.5	0.149
		Moderate	1	0.54 (−0.97 to 2.05)	0.482	-	-
		High	1	−0.63 (−1.14 to −0.12)	0.016	-	-
	Chicken	Low	1	7.10 (6.53 to 7.67)	<0.001	-	-
		Moderate	1	4.50 (3.99 to 5.01)	<0.001	-	-
		High	1	5.10 (4.57 to 5.63)	<0.001	-	-
Water	Pigs	Low	1	0.12 (−0.08 to 0.32)	0.251	-	-
		Moderate	1	0.61 (0.18 to 1.04)	0.006	-	-
		High	1	0.90 (0.25 to 1.55)	0.007	-	-
Crude ash	Pigs	Low	1	−0.28 (−0.49 to −0.07)	0.008	-	-
		Moderate	1	−0.58 (−1.02 to −0.14)	0.009	-	-
		High	1	−1.01 (−1.56 to −0.46)	<0.001	-	-
	Chicken	Low	1	1.90 (0.50 to 3.30)	0.008	-	-
		Moderate	1	−0.50 (−1.50 to 0.50)	0.326	-	-
		High	1	3.60 (2.46 to 4.74)	<0.001	-	-
Crude fat	Pigs	Low	1	0.07 (−0.12 to 0.26)	0.468	-	-
		Moderate	1	0.05 (−0.40 to 0.50)	0.828	-	-
		High	1	−0.43 (−0.87 to 0.01)	0.056	-	-

WMD, mean differences; CI, confidence interval.

**Table 6:** The summary results of mulberry leaf powders on serum antioxidant capacity and immune organ indexes

Outcome	Animal species	Intervention	No of studies	WMD and 95%CI	p-value	*I* ^2^	Q statistic
Catalase activity	Chicken	Low	1	0.82 (0.40 to 1.24)	<0.001	-	-
		Moderate	1	1.62 (1.23 to 2.01)	<0.001	-	-
		High	1	1.85 (1.39 to 2.31)	<0.001	-	-
Total antioxidant capacity	Chicken	Low	1	0.14 (0.00 to 0.28)	0.043	-	-
		Moderate	1	0.48 (0.35 to 0.61)	<0.001	-	-
		High	1	0.21 (0.07 to 0.35)	0.003	-	-
Superoxide dismutase	Chicken	Low	1	3.91 (0.67 to 7.15)	0.018	-	-
		Moderate	1	9.68 (7.01 to 12.35)	<0.001	-	-
		High	1	14.22 (11.03 to 17.41)	<0.001	-	-
Glutathione peroxidase	Chicken	Low	1	7.38 (3.94 to 10.82)	<0.001	-	-
		Moderate	1	17.89 (14.07 to 21.71)	<0.001	-	-
		High	1	21.89 (18.24 to 25.54)	<0.001	-	-
Malondialdehyde	Chicken	Low	1	−−0.72 (−1.19 to −0.25)	0.003	-	-
		Moderate	1	−1.45 (−1.92 to −0.98)	<0.001	-	-
		High	1	−1.71 (−2.29 to −1.13)	<0.001	-	-
IL-1β	Pigs	Low	1	54.03 (50.18 to 57.88)	<0.001	-	-
		High	1	8.48 (4.65 to 12.31)	<0.001	-	-
IL-2	Pigs	Low	1	97.90 (94.24 to 101.56)	<0.001	-	-
		High	1	62.88 (59.40 to 66.36)	<0.001	-	-
IL-6	Pigs	Low	1	8.22 (6.59 to 9.85)	<0.001	-	-
		High	1	16.88 (16.04 to 17.72)	<0.001	-	-
IL-8	Pigs	Low	1	48.89 (43.93 to 53.86)	<0.001	-	-
		High	1	8.96 (3.71 to 14.21)	0.001	-	-
INF-γ	Pigs	Low	1	5.55 (4.77 to 6.33)	<0.001	-	-
		High	1	7.86 (7.11 to 8.61)	<0.001	-	-
Thymus index	Pigs	Low	1	0.35 (0.05 to 0.65)	0.024	-	-
		High	1	0.56 (0.15 to 0.97)	0.008	-	-
Spleen index	Pigs	Low	2	0.21 (0.06 to 0.36)	0.005	0.0	0.934
		High	1	0.21 (0.07 to 0.35)	0.004	-	-
IgM	Pigs	Low	1	0.04 (−0.09 to 0.17)	0.550	-	-
		High	1	−0.09 (−0.15 to −0.03)	0.003	-	-
IgG	Pigs	Low	1	1.01 (0.47 to 1.55)	<0.001	-	-
		High	1	−0.27 (−0.96 to 0.42)	0.443	-	-

WMD, mean differences; CI, confidence interval.

**Table 7:** The summary results of mulberry leaf powders on egg quality

Outcome	Animal species	Intervention	No of studies	WMD and 95%CI	p-value	*I* ^2^	Q statistic
Egg weight	Chicken	Low	2	−0.24 (−1.48 to 1.00)	0.702	0.0	0.840
		Moderate	1	0.27 (−1.26 to 1.80)	0.730	-	-
		High	2	−0.05 (−1.36 to 1.25)	0.937	0.0	0.749
Eggshell strength	Chicken	Low	1	2.91 (1.31 to 4.51)	<0.001	-	-
		Moderate	1	3.52 (1.85 to 5.19)	<0.001	-	-
		High	1	4.85 (3.24 to 6.46)	<0.001	-	-
Yolk color	Chicken	Low	1	0.61 (0.32 to 0.90)	<0.001	-	-
		Moderate	1	0.77 (0.47 to 1.07)	<0.001	-	-
		High	1	0.94 (0.71 to 1.17)	<0.001	-	-
Eggshell thickness	Chicken	Low	1	0.02 (0.01 to 0.03)	<0.001	-	-
		Moderate	1	0.02 (0.01 to 0.03)	<0.001	-	-
		High	1	0.00 (−0.01 to 0.01)	1.000	-	-
Egg shape index	Chicken	Low	1	0.01 (−0.07 to 0.09)	0.794	-	-
		Moderate	1	−0.03 (−0.10 to 0.04)	0.414	-	-
		High	1	−0.01 (−0.07 to 0.05)	0.757	-	-
Haugh unit	Chicken	Low	1	0.75 (−1.50 to 3.00)	0.514	-	-
		Moderate	1	1.07 (−1.31 to 3.45)	0.379	-	-
		High	1	0.89 (−1.18 to 2.96)	0.399	-	-
Egg yolk weight	Chicken	Low	1	−0.31 (−0.99 to 0.37)	0.373	-	-
		Moderate	1	−0.10 (−0.75 to 0.55)	0.763	-	-
		High	1	0.10 (−0.55 to 0.75)	0.764	-	-
Eggshell weight	Chicken	Low	1	0.08 (−0.14 to 0.30)	0.471	-	-
		Moderate	1	0.21 (0.02 to 0.40)	0.032	-	-
		High	1	0.07 (−0.15 to 0.29)	0.534	-	-
Protein height	Chicken	Low	1	−0.14 (−0.75to 0.47)	0.653	-	-
		Moderate	1	0.15 (−0.36 to 0.66)	0.564	-	-
		High	1	0.30 (−0.19 to 0.79)	0.232	-	-
Egg yolk ratio	Chicken	Low	1	−1.34 (−1.65 to −1.03)	<0.001	-	-
		Moderate	1	−0.69 (−1.01 to −0.37)	<0.001	-	-
		High	1	−0.52 (−0.81 to −0.23)	<0.001	-	-
Egg yolk moisture content	Chicken	Low	1	0.09 (−0.9 to 1.17)	0.870	-	-
		Moderate	1	−1.82 (−3.43 to −0.21)	0.026	-	-
		High	1	−4.43 (−6.71 to −2.15)	<0.001	-	-

WMD, mean differences; CI, confidence interval.

## Data Availability

The datasets used and/or analyzed during the current study are available from the corresponding author on reasonable request.
